# Mechanisms and clinical landscape of N6-methyladenosine (m6A) RNA modification in gastrointestinal tract cancers

**DOI:** 10.1007/s11010-024-05040-x

**Published:** 2024-06-10

**Authors:** Dan-Hua Zhu, Kun-Kai Su, Xiao-Xi Ou-Yang, Yan-Hong Zhang, Xiao-Peng Yu, Zu-Hong Li, Seyedeh-Sara Ahmadi-Nishaboori, Lan-Juan Li

**Affiliations:** 1 State Key Laboratory for Diagnosis and Treatment of Infectious Diseases, National Clinical Research Center for Infectious Diseases, National Medical Center for Infectious Diseases, Collaborative Innovation Center for Diagnosis and Treatment of Infectious Diseases, The First Affiliated Hospital, Zhejiang University School of Medicine, Hangzhou, 310003 China; 2 International School of Medicine, Zhejiang University, Hangzhou, 311121 China

**Keywords:** Gastrointestinal tract cancers, m6A, Expression level, Mechanism, Clinical applications

## Abstract

Epigenetics encompasses reversible and heritable chemical modifications of non-nuclear DNA sequences, including DNA and RNA methylation, histone modifications, non-coding RNA modifications, and chromatin rearrangements. In addition to well-studied DNA and histone methylation, RNA methylation has emerged as a hot topic in biological sciences over the past decade. N6-methyladenosine (m6A) is the most common and abundant modification in eukaryotic mRNA, affecting all RNA stages, including transcription, translation, and degradation. Advances in high-throughput sequencing technologies made it feasible to identify the chemical basis and biological functions of m6A RNA. Dysregulation of m6A levels and associated modifying proteins can both inhibit and promote cancer, highlighting the importance of the tumor microenvironment in diverse biological processes. Gastrointestinal tract cancers, including gastric, colorectal, and pancreatic cancers, are among the most common and deadly malignancies in humans. Growing evidence suggests a close association between m6A levels and the progression of gastrointestinal tumors. Global m6A modification levels are substantially modified in gastrointestinal tumor tissues and cell lines compared to healthy tissues and cells, possibly influencing various biological behaviors such as tumor cell proliferation, invasion, metastasis, and drug resistance. Exploring the diagnostic and therapeutic potential of m6A-related proteins is critical from a clinical standpoint. Developing more specific and effective m6A modulators offers new options for treating these tumors and deeper insights into gastrointestinal tract cancers.

## Introduction

Gastrointestinal tract cancers are malignant diseases caused by multiple factors, with high incidence and mortality rates worldwide. They mainly include liver, gastric, colorectal, and pancreatic cancers [1–4]. Early diagnosis and patient prognosis remain formidable challenges due to the subtle symptoms at onset and during tumor invasion and metastasis [5–7]. Advances in medical science have led to the widespread use of diagnostic techniques such as endoscopy, tissue biopsy, and imaging technology for the diagnosis and management of gastrointestinal tract cancers [8, 9]. These essential tools allow the identification of the precise location and severity of the specific lesions [10–13]. Nearly all gastrointestinal tract cancers exhibit genomic and epigenomic DNA alterations, which, along with other microenvironment factors, play a crucial role in initiating and driving the progression of cancers [14–19]. Therefore, understanding the molecular mechanisms of tumorigenesis remains essential for improved diagnosis and treatment.

With the advancement of specific antibodies and high-throughput sequencing technologies, researchers can investigate N6-methyladenosine (m6A) sites in greater depth, marking an important milestone in RNA epitranscriptomics [20–24]. This reversible modification is regulated by the balance of "writers" and "erasers" proteins, indicating the potential of these proteins in regulating biological processes [25–28]. The core members of the highly conserved mRNA methyltransferase complex, known as the m6A "writer" complex, include methyltransferase-like (METTL)3, METTL14, and Wilms’ tumor 1-associating protein (WTAP) [29–31]. m6A modification attracts "reader" proteins to exert their biological functions. These proteins can be categorized into three types based on structural domains. The first type features the conserved YTH (YT521-B homology) domain, while the second type includes heterologous nuclear ribonucleoproteins (hnRNP) such as hnRNPC, hnRNPG, and hnRNPA2B1 [32, 33]. The third type of m6A "reader" protein includes insulin-like growth factor-binding protein (IGFBP) family proteins, such as IGFBP1-3 [34–38]. "Reader" proteins regulate almost every aspect of RNA metabolism, including stability, translation, and splicing of m6A transcript products [37, 39, 40]. Finally, m6A modification can be reversed through enzymatic reactions mediated by alpha-ketoglutarate dependent dioxygenase (FTO) and AlkB homolog 5 (ALKBH5), known as m6A "erasers" [41, 42]. m6A modification plays an important role in RNA epitranscriptomics, and its regulation and function are closely related to the interaction of multiple proteins affecting RNA stability, translation, degradation, nuclear localization, and splicing [43–48]. Deciphering these processes is crucial for understanding gene expression regulation mechanisms and disease development [49–54].

Gastrointestinal tract cancers are common malignant tumors with complex pathogenesis. Specifically, m6A methylation participates in the occurrence and development of gastrointestinal tract cancers by influencing several biological processes such as tumor cell proliferation, apoptosis, invasion, metabolism, immune response, and metastasis [55–58]. Furthermore, changes in m6A methylation levels are closely associated with the clinical and pathological features of gastrointestinal tract cancer. Elevated m6A methylation levels are closely correlated with poor clinical prognosis in liver cancer [59–61]. In gastric cancer, increased m6A methylation levels are reported to facilitate tumor cell proliferation and invasion while reducing patients' survival [62]. Therefore, m6A methylation has emerged as a potential diagnostic and prognostic marker or a promising therapeutic target [49]. Current studies are developing diagnostic and prognostic methods for different subtypes of gastrointestinal tract cancers, targeting m6A methylation to guide individualized treatment [63–66]. Combined upregulation of METTL3 and YTHDF1 was validated as a biological marker reflecting the malignancy level of liver cancer and patient prognosis [63]. Additionally, ongoing research aims to develop m6A methylation-targeted treatments to prolong patient survival and relieve cancer symptoms [67]. Further understanding and in-depth research into this modification will expand its application prospects as a novel diagnostic, prognostic indicator, and therapeutic target in gastrointestinal tract cancers [68, 69].

This review aims to explore the correlation between m6A methylation changes, clinical and pathological features, the role of m6A methylation in cancer, and its involvement in cancer processes. Summarizing these findings provides a strong foundation for considering m6A methylation as a new target for developing clinical strategies for the management of gastrointestinal tract cancers.

## Molecular mechanisms of m6A modification in gastrointestinal tract cancers

**Fig. 1:**
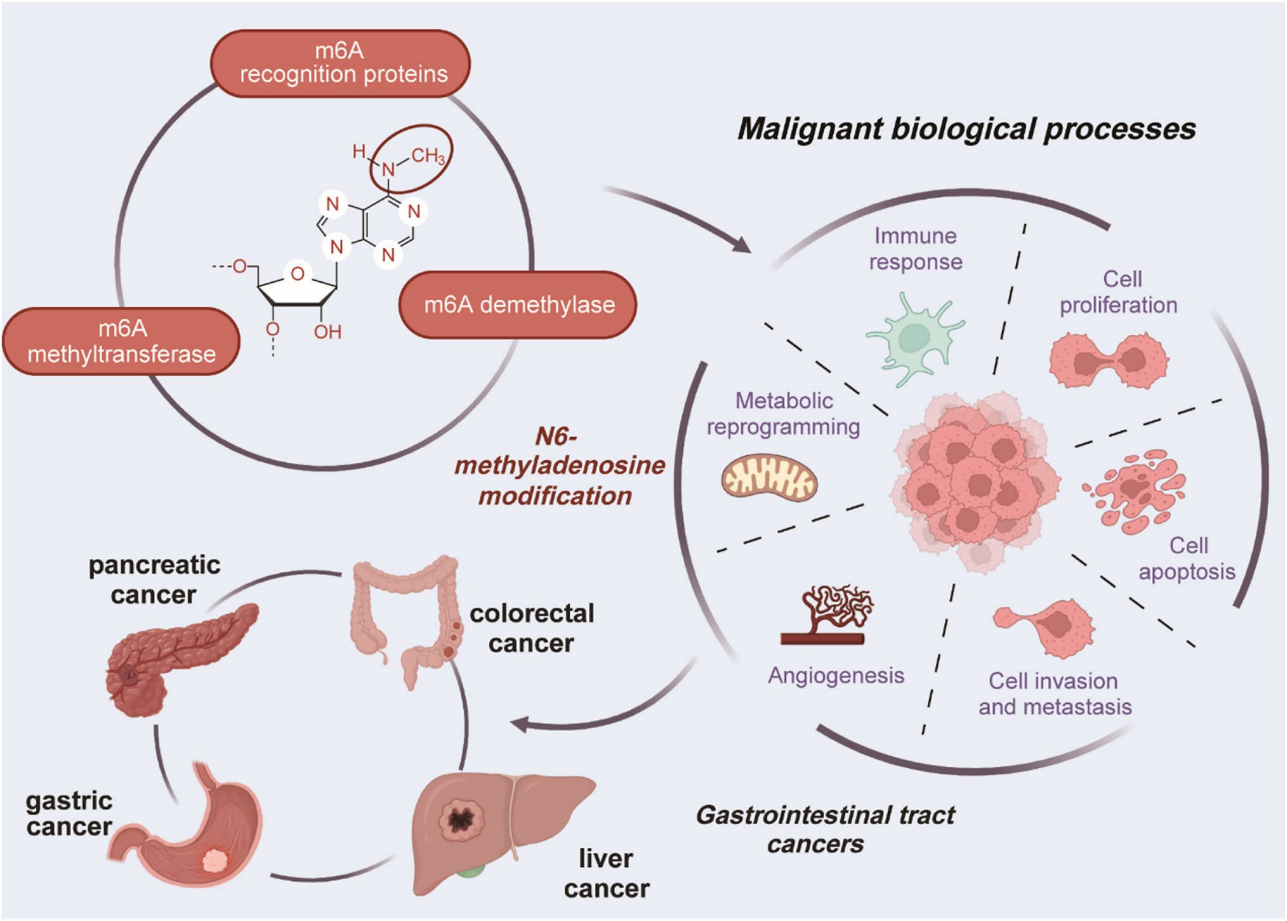
Overview of the involvement of m6a in several types of common gastrointestinal tract cancers. The dysregulation of m6A regulators plays essential roles in gastrointestinal tract cancers by impacting various biological processes such as cell proliferation, migration, invasion, and metabolic reprogramming

m6A RNA modification is a reversible process involved in the regulation of mRNA stability, splicing, translation, and other processes [70]. Increasing evidence links m6A modification to cancer progression, as it promotes tumor cell growth, survival, and invasion, facilitates the maintenance of stem cell self-renewal and differentiation, and confers resistance to radiotherapy and chemotherapy (Fig. 1) [71–74].

Furthermore, m6A has dual functions in cancer [75]. Its impact on the regulation of target genes depends on three key factors [76, 77]. Firstly, it depends on whether m6A-regulated target genes are oncogenes or tumor suppressor genes. Secondly, it is influenced by the role of the aberrant m6A regulators in cancer as "writers", "erasers", or "readers". Furthermore, the ultimate effect of these "readers" on target genes varies depending on their specific interactions, either promoting or inhibiting RNA expression [45, 78, 79]. Gastrointestinal tract cancers exhibit altered expression levels of m6A regulators, leading to dysregulated m6A modification and aberrant gene expression [80]. Exploring m6A modifications in gastrointestinal tract cancers reveals a new mechanism underlying tumor progression, with promising perspectives for clinical applications [81].

**Fig. 2:**
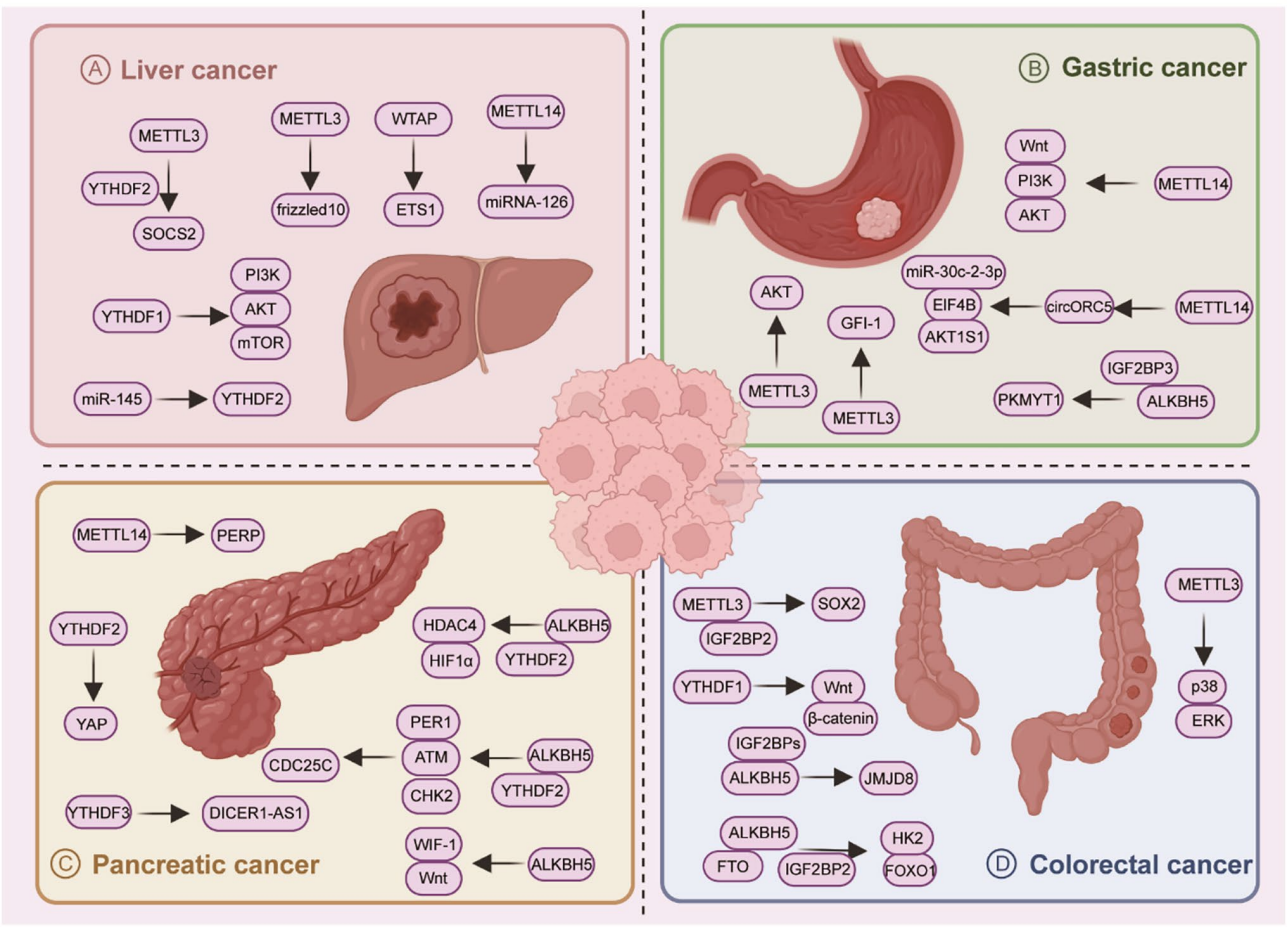
The main functions and mechanism of m6A in several types of common gastrointestinal tract cancers. Various m6A regulators modulate expression levels of cancer-related genes by influencing RNA stability, post-transcriptional modifications, and translation efficiency, thus participating in the occurrence and development of gastrointestinal tract cancers

In this section, we focus on m6A methylation level changes in common digestive tract tumors, including liver, gastric, colorectal, and pancreatic cancers (Table 1). Additionally, we elucidate how m6A regulates biological processes and drives tumor progression by influencing target gene expression (Fig. 2).

**Table 1:** The roles of RNA m6A in gastrointestinal tract cancers

Cancer type	m6A regulators	Function	Regulation	Role	Target	Upstream	Reader	Functions	Involved pathway	References
Liver cancer	METTL3	Writers	Upregulation	Oncogene	SOCS2	/	YTHDF2	Promote cell proliferation, migration, and colony formation	METTL3/YTHDF2/SOCS2	[99]
Liver cancer	METTL3	Writers	Upregulation	Oncogene	frizzled10	/	/	Promote self-renewal and expansion of liver cancer stem cells and metastasis of liver cancer cells	Frizzled10/β-catenin/c-Jun	[98]
Liver cancer	METTL14	Writers	Downregulation	Tumor suppressor	miRNA- 126	/	/	Inhibit tumor metastasis	METTL14/miRNA-126	[101]
Liver cancer	WTAP	Writers	Upregulation	Oncogene	ETS1	/	/	Promote cell proliferation capability and tumor growth	WTAP/ETS1	[100]
Liver cancer	YTHDF1	Reader	Upregulation	Oncogene	PI3K/AKT/mTOR signaling pathway	/	/	Promote cell proliferation, cell cycle progression, migration, invasion, and epithelial-mesenchymal transition	YTHDF1/PI3K/AKT /mTOR	[102]
Liver cancer	YTHDF2	Reader	Upregulation	Oncogene	/	miR-145	/	Promote cell proliferation	miR-145/YTHDF2	[103]
Gastric cancer	METTL3	Writers	Upregulation	Oncogene	AKT	/	/	Promote cell proliferation and mobility	METTL3/AKT	[123]
Gastric cancer	METTL3	Writers	Upregulation	Oncogene	GFI-1	/	/	Promote cell proliferation, migration, and epithelial-mesenchymal transition	METTL3/GFI-1	[122]
Gastric cancer	METTL14	Writers	Downregulation	Tumor suppressor	Wnt/PI3K/AKT signaling	/	/	Inhibit cell proliferation and invasiveness	METTL14/Wnt/PI3K /AKT	[124]
Gastric cancer	METTL14	Writers	Downregulation	Tumor suppressor	circORC5	/	/	Inhibit cell growth and invasion	METTL14/circORC5 /miR-30c-2-3p/ElF4B /AKT1S1	[141]
Gastric cancer	ALKBH5	Eraser	Downregulation	Tumor suppressor	PKMYT1	/	IGF2BP3	Suppress cell invasion	ALKBH5/IGF2BP3/PKMYT1	[125]
Colorectal cancer	METTL3	Writers	Upregulation	Oncogene	SOX2	/	IGF2BP2	Promote cell self-renewal, stem cell frequency, and migration	METTL3/IGF2BP2/SOX2	[136]
Colorectal cancer	METTL3	Writers	Downregulation	Tumor suppressor	p38/ERK pathway	/	/	Inhibit cell proliferation and migration	METTL3/p38/ERK	[137]
Colorectal cancer	YTHDF1	Reader	Upregulation	Oncogene	Wnt/β-catenin pathway	/	/	Promote tumorigenicity and cancer stem cell-like activity	YTHDF1/Wnt/β-catenin	[138]
Colorectal cancer	ALKBH5	Eraser	Downregulation	Tumor suppressor	JMJD8	/	IGF2BPs	Suppress tumor cell glycolysis	ALKBH5/IGF2BPs/JMJD8	[140]
Colorectal cancer	FTO and ALKBH5, IGF2BP2	Eraser, reader	Downregulation	Tumor suppressor	HK2	/	IGF2BP2	Inhibit cell glycolysis	FTO/ALKBH5/IGF2BP2 /HK2/FOXO1	[139]
Pancreatic cancer	METTL14	Writers	Upregulation	Oncogene	PERP	/	/	Promote cell proliferation and migration	METTL14/PERP	[155]
Pancreatic cancer	YTHDF2	Reader	Upregulation	Oncogene	YAP	/	YTHDF2	Promote cell proliferation and inhibit cell migration, invasion, and epithelial-mesenchymal transition	YTHDF2/YAP	[157]
Pancreatic cancer	YTHDF3	Reader	Upregulation	Oncogene	DICER1-AS1	/	/	Promote cell glycolytic metabolism and migration	YTHDF3/DICER1-AS1	[161]
Pancreatic cancer	ALKBH5	Eraser	Upregulation	Oncogene	HDAC4	/	YTHDF2	Promote cell glycolytic metabolism and migration	ALKBH5/YTHDF2/HDAC4 /HIF1α	[160]
Pancreatic cancer	ALKBH5	Eraser	Downregulation	Tumor suppressor	PER1	/	YTHDF2	Reduce cell proliferative, migrative, and invasive activities	ALKBH5/YTHDF2/PER1 /ATM/CHK2/P53/CDC25C	[158]
Pancreatic cancer	ALKBH5	Eraser	Downregulation	Tumor suppressor	WIF-1	/	/	Inhibit cell proliferation, migration, and invasion	ALKBH5/WIF-1/Wnt	[159]

### Liver cancer

Liver cancer is a common malignancy that is often discovered in advanced stages due to the lack of early symptoms. Hepatocellular carcinoma (HCC) ranks as the fourth leading cause of cancer-related deaths globally [82–85]. Early-stage HCC benefits significantly from surgical resection, but late-stage diagnosis hampers the feasibility and effectiveness of surgery [86–88]. Molecular targeted therapy and immune checkpoint inhibitors are pivotal for treating advanced HCC [89]. Drugs such as sorafenib, lenvatinib, and nivolumab have been widely used in clinical practice [90–92]. However, their usage faces several limitations, including high costs [93–96].

Therefore, in terms of future diagnosis and treatment, it is important to deeply understand the molecular mechanisms of m6A regulators in liver cancer development and develop highly effective, low side-effect treatments to mitigate this disease [97]. Research has shown that METTL3 mediates frizzled10 activation, initiating the β-catenin/YAP1 axis in HCC cells. In turn, the frizzled 10-β-catenin/c-Jun axis also transcriptionally activates METTL3 expression, forming a positive feedback loop that promotes self-renewal, expansion of liver cancer stem cells, and metastasis of liver cancer cells [98]. METTL3, as an oncogene, when knocked down, significantly reduces the proliferation, migration, and colony formation of HCC cells due to the YTHDF2-dependent m6A modification on suppressor of cytokine signaling 2 (SOCS2) mRNA. This change increases the expression of SOCS2 [99]. High expression of WTAP was revealed to suppress the expression of ETS proto-oncogene 1 (ETS1) through the m6A-HuR pattern, reducing the expression of tumor suppressors p21 and p27 and promoting the proliferation of HCC cells and tumor growth [100].

Additionally, METTL14 and microprocessor protein DiGeorge Critical Region 8 collaborate to promote the processing of miR-126, suppressing HCC metastasis through an m6A-dependent mechanism [101]. High expression of YTHDF1 enhances the activation of the phosphatidylinositol 3-kinase (PI3K)/protein kinase B (AKT)/mammalian target of rapamycin (mTOR) signaling pathway, promoting proliferation, cell cycle progression, migration, invasion, and epithelial-mesenchymal transition of liver cancer cells [102]. miR-145, a suppressive non-coding RNA in various tumors, downregulates the expression of YTHDF2 and elevates the mRNA m6A level in HCC HepG2 cells, thereby inhibiting the proliferation of HCC cells [103].

### Gastric cancer

Gastric cancer is ranked the fifth most common malignancy and the third leading cause of cancer-related mortality in the world [104–107]. Risk factors encompass environmental and hereditary factors, including genetic mutations, chromosomal abnormalities, differential gene expression, and epigenetic changes [108–113]. Environmental factors include Helicobacter pylori infection, age, high salt intake, and low intake of fruits and vegetables [114, 115]. Despite a global decline in the incidence of gastric cancer over the past century, the median survival time for advanced gastric cancer is still less than one year due to late-stage detection [116–119]. Early detection and treatment remain critical for improving patient survival rates. Recent studies have revealed a significant increase in m6A methylation levels of total RNA in gastric cancer.

Dysregulation of METTL3 contributes to the oncogenesis of gastric cancer as METTL3 regulates the translation of oncogenes [120, 121]. In 415 patients from The Cancer Genome Atlas (TCGA) cohort, METTL3 expression was increased in gastric cancer tissues and associated with poor patient prognosis. Additionally, METTL3 deficiency reduced the expression level of growth factor independence 1 (GFI-1). Knockdown of METTL3 significantly inhibited proliferation, migration, and progression of epithelial-mesenchymal transition in gastric cancer cells [122].

The AKT signaling pathway is an important cellular signaling pathway that promotes the proliferation, migration, and invasion of gastric cancer cells. When METTL3 is downregulated, the AKT signaling pathway cannot be activated properly, inhibiting the proliferation, migration, and invasion ability of gastric cancer cells [123]. Consequently, knocking down METTL14 in vitro activates the Wingless/Integrated (Wnt) and PI3K/Akt signaling pathways, promoting the proliferation and invasion of gastric cancer cells [124].

Demethylase ALKBH5 inhibited the metastatic ability of gastric cancer cells through negative regulation of the expression of protein kinase, membrane-associated tyrosine/threonine 1 (PKMYT1), a member of the serine/threonine protein kinase family. Targeting IGF2BP3 in gastric cancer HGC-27 cells resulted in a significant downregulation of PKMYT1 expression, highlighting the potential role of the ALKBH5/PKMYT1/IGF2BP3 regulatory signaling pathway in gastric cancer metastasis [125].

### Colorectal cancer

In recent years, the heterogeneity of colorectal cancer has attracted increasing attention [126–129]. Although substantial progress has been made in anti-tumor strategies for colorectal cancer, including multidisciplinary treatment, microbe-based therapies, molecular targeted therapy, and immunotherapy, many patients are still diagnosed at advanced stages [130–132]. Early diagnosis and personalized treatment remain crucial for addressing this disease. Therefore, identifying sensitive biomarkers for prognosis, recurrence monitoring, and individualized treatment management of colorectal cancer is of utmost importance [133–135]. METTL3 has been shown to play a complex role by participating in various regulatory pathways. It is highly expressed and mediates methylation modification of SRY (sex determining region Y)-box 2 (SOX2), preventing SOX2 mRNA degradation via IGF2BP2 recognition and contributing to cellular self-renewal, migration, and metastasis [136]. However, METTL3 can also activate the p38/ extracellular signal-regulated kinase (ERK) pathway, inhibiting the proliferation and migration of colorectal cancer cells [137].

YTHDF1 is highly expressed in various tumors and is closely associated with oncogenesis. Silencing the expression of YTHDF1 significantly suppresses the activity of the Wnt/β-catenin pathway, inhibiting tumor formation and stem-like activity in colorectal cancer cells [138]. Extensive research has revealed that m6A regulators play a crucial role in regulating abnormal metabolism in tumor cells. In colorectal cancer under high-fat conditions, ALKBH5 and FTO downregulation increases m6A methylation on HK2 mRNA through IGF2BP2. These regulators further activate the class O of forkhead box transcription factors (FOXO) signaling pathway, accelerating glycolysis, proliferation, and tumor progression [139]. Molecular investigations indicate that ALKBH5 collaborates with METTL14/IGF2BPs to suppress tumor cell glycolysis by negatively regulating the Jumonji domain-containing protein 8 (JMJD8)/pyruvate kinase M2 (PKM2) signaling axis, slowing down colorectal cancer progression [140]. m6A modification of circRNAs is also critical for regulating the progression of gastric cancer. Knockdown of METTL14 reduces the circORC5 m6A levels, increasing circORC5 expression and inhibiting tumor progression through the miR-30c-2-3p/AKT1 substrate 1 (AKT1S1) axis [141].

### Pancreatic cancer

Pancreatic cancer has a high global incidence and mortality rate and is often diagnosed at an advanced stage, due to nonspecific early symptoms [142–144]. Consequently, its prognosis is unfavorable, and it has limited treatment options. Surgery is most effective for localized cases but is often inapplicable due to late diagnosis [145, 146]. Despite therapeutic advancements such as molecular targeted therapy and immunotherapy, pancreatic cancer still has a low five-year survival rate of approximately 10% [147, 148]. Ongoing research focuses on understanding in-depth molecular mechanisms for early detection, innovative therapies, and personalized management [149–152]. Recent studies have highlighted the significant role of m6A regulators in the tumorigenesis and progression of pancreatic cancer [153, 154]. Elevated METTL14 expression significantly enhanced pancreatic cancer cell proliferation and migration by directly targeting downstream p53 effector related to PMP-22 (PERP) mRNA in an m6A-dependent way [155, 156].

The overexpressed YTHDF2 binds to m6A-modified yes-associated protein 1 (YAP) mRNA, inhibiting YAP expression and promoting the migration-proliferation dichotomy and epithelial-mesenchymal transition of pancreatic cancer cells [157]. ALKBH5 activates Period1 (PER1) gene through m6A demethylation and in an m6A-YTHDF2-dependent manner, reactivating the Ataxia telangiectasia mutated/checkpoint kinase 2/tumor protein 53/cell division cycle 25C signaling pathway and inhibiting the proliferation, migration, and invasiveness of pancreatic cancer cells [158]. ALKBH5 also promotes Wnt inhibitory factor 1 (WIF-1) transcription, suppressing Wnt signaling in pancreatic cancer cells and inhibiting cancer progression [159].

Under hypoxia, the significantly increased transcription and protein levels of ALKBH5 in pancreatic cancer enhance glycolysis and cell migration. ALKBH5 controls m6A modification of histone deacetylase type 4 (HDAC4) recognized by YTHDF2, thereby upregulating HDAC4 expression. Upregulated HDAC4 stabilizes hypoxia-inducible factor-1α (HIF1α) in hypoxic pancreatic cancer cells, creating a positive feedback loop that increases ALKBH5 expression [160]. Additionally, under glucose deprivation, miR-5586-5p induces the overexpression of YTHDF3, leading to m6A modification of DICER1 antisense RNA1 and promoting cell glycolysis, tumor growth, and metastasis in pancreatic cancer [161].

## Clinical applications of m6A modification in gastrointestinal tract cancers

**Fig. 3:**
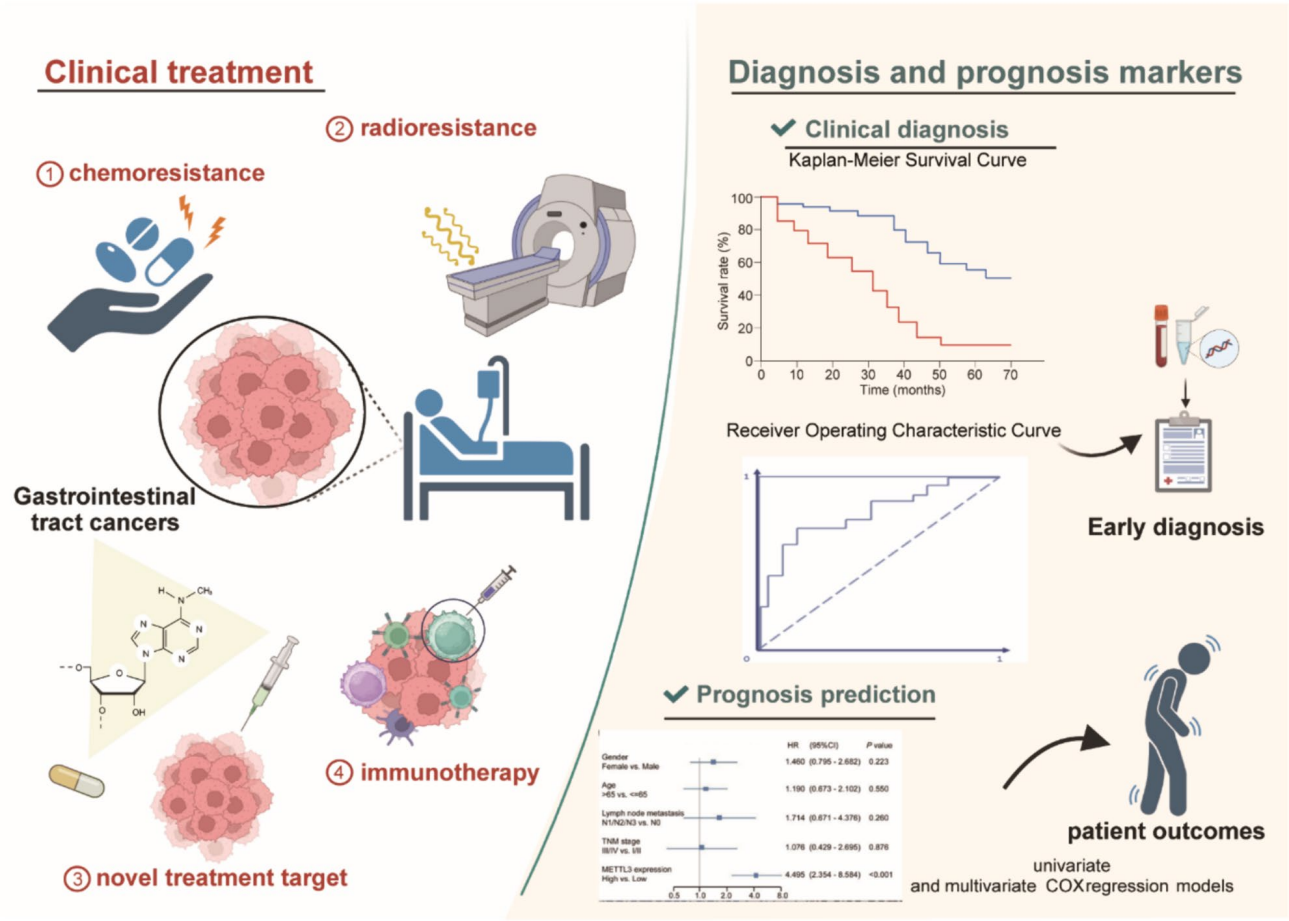
m6A modification holds promise in gastrointestinal tract cancers for clinical diagnosis, prognosis prediction, and treatment guidance. Kaplan–Meier survival and receiver operating characteristic curves confirmed the reliability of abnormal m6A levels as diagnostic indicators. Univariate and multivariate regression models support m6A as an independent prognostic indicator. Research on m6A regulation mechanisms in the progression of digestive tract tumors suggests that targeting abnormal m6A levels improves chemotherapy and radiotherapy resistance. Modulating m6A levels enhances the efficacy of immunotherapy, improving patient survival rates. Furthermore, targeted drug delivery systems achieve significant anticancer effects

Many studies have elaborated on the significance of m6A regulators in gastrointestinal tract cancers, particularly focusing on their roles as diagnostic, prognostic indicators, and potential therapeutic targets. (Fig. 3) [162–165]. Increasing evidence links changes in m6A modification levels to clinical characteristics in various digestive tract tumors, providing new possibilities for non-invasive early diagnosis of malignant gastrointestinal tract cancers (Table 2) [61, 166, 167].

**Table 2:** Clinical applications of m6A modification in gastrointestinal tract cancers

M6A regulators	Related targets	Clinical applications	Disease	Regulation	Year	References
METTL3	lncRNA ARHGAP5-AS1	Chemoresistance	Gastric cancer	Upregulation	2019	[185]
METTL3	/	Chemo- and radio-resistance	Pancreatic cancer	Upregulation	2018	[187]
METTL3	frizzled10	Chemoresistance	Liver cancer	Upregulation	2023	[98]
YTHDF1	c-Myc	Chemoresistance	Colorectal cancer	Upregulation	2017	[194]
YTHDF1	frizzled7, and Wnt/β-catenin pathway	Immunotherapy	Gastric cancer	Upregulation	2023	[195]
IGF2BP1	ABL	Chemoresistance	Gastric cancer	Upregulation	2022	[188]
ALKBH5	WIF-1	Chemoresistance	Pancreatic cancer	Downregulation	2020	[159]
ALKBH5	DDIT4-AS1	Chemoresistance	Pancreatic cancer	Downregulation	2022	[189]
ALKBH5	JMJD8	ALKBH5 mRNA nanotherapeutics	Colorectal cancer	Downregulation	2023	[140]
METTL3	HDGF	A predictive prognostic marker	Gastric cancer	Upregulation	2020	[175]
METTL3 and YTHDF1	/	A predictive prognostic marker	Hepatocellular carcinoma (HCC)	Upregulation	2019	[63]
YTHDF1	/	A predictive prognostic marker	Hepatocellular carcinoma (HCC)	Upregulation	2018	[65]
IGF2BPs	/	A predictive prognostic marker	Gastric cancer	Upregulation	2022	[170]
ALKBH1 and FTO	/	A predictive prognostic marker	Gastric cancer	Downregulation	2019	[64]
ALKBH5	PER1	A predictive prognostic marker	Pancreatic cancer	Downregulation	2020	[158]
ALKBH5	/	A predictive prognostic marker	Pancreatic cancer	Downregulation	2018	[171]
ALKBH5 and FTO	/	A diagnostic biomarker	Gastric cancer	Downregulation	2020	[169]

## m6A modification as diagnostic and prognostic indicators in gastrointestinal tract cancers

Circulating tumor cells (CTCs), which are cells shed from tumors into the bloodstream, offer a non-invasive method for diagnosing and monitoring cancer [168]. Elevated levels of m6A modification in CTCs compared to whole blood samples suggest its potential as a diagnostic marker for cancer, particularly in gastrointestinal tract cancers. For example, the level of m6A in peripheral blood RNA was significantly higher in patients with gastric cancer than in patients with benign gastric diseases and healthy controls. The study also assessed m6A levels in peripheral blood RNA as a non-invasive diagnostic biomarker for patients with gastric cancer using a receiver operating characteristic (ROC) curve analysis. ROC analysis revealed an area under the curve of 0.929, outperforming commonly used biomarkers such as carcinoembryonic antigen and carbohydrate antigen 199. This indicated that the m6A levels in peripheral blood RNA have high accuracy in diagnosing gastric cancer [169, 170]. In pancreatic cancer, m6A levels showed promise as a predictive and prognostic marker. Reduced ALKBH5 levels in pancreatic cancer tissues were identified through various technologies, and survival analysis indicated a significant association between ALKBH5 expression and poor prognosis in patients with pancreatic cancer [158, 171].

Furthermore, multiple m6A regulators, including METTL3 and ALKBH5, have been identified as independent prognostic indicators for gastrointestinal tract cancers through multivariate Cox regression analysis and Kaplan–Meier survival curves [172–174]. High expression of METTL3 in gastric cancer is associated with lymph node metastasis and advanced TNM stage. It serves as an adverse prognostic indicator for patient survival and recurrence. Multivariate Cox regression analysis has further demonstrated that METTL3 is an independent prognosis predictor in patients with gastric cancer. When combined with clinical risk score (TNM stage), METTL3 detection significantly improves prognostic accuracy, underlining its clinical significance in gastric cancer [175]. High expression of METTL3 may serve as an adverse prognostic indicator for patient survival and recurrence. Numerous studies have also analyzed large TCGA datasets to investigate the relationship between abnormal expression of m6A regulators, patient prognosis, and tumor staging in gastrointestinal tract cancers [176, 177]. Robust m6A-related prognostic models and risk-scoring systems have been developed using clinical follow-up data and public databases like TCGA, aiding in predicting disease progression and survival status. Moreover, downregulation of ALKBH5 is correlated with poor prognosis in colorectal cancer patients. Chen Rui's team detected a significant downregulation of ALKBH5 in 1,078 patients with colorectal cancer tissues compared to healthy controls in a ten-year follow-up cohort and the TCGA dataset. This downregulation was associated with poor prognosis in patients with colorectal cancer [140]. In addition, researchers have also developed robust m6A-related prognostic models and risk-scoring systems using clinical follow-up data and public databases such as TCGA. These models and scoring systems can individually evaluate the prognosis and survival of patients with gastrointestinal tract cancers based on their m6A regulator expression and other clinical characteristics, better-predicting disease progression and survival status and providing important references for clinical decision-making [172, 178–181].

## Therapeutic potential of m6A modification in gastrointestinal tract cancers

Various m6A regulators participate in carcinogenic processes in gastrointestinal tract cancers, such as cell proliferation, migration, invasion, stem cell characteristics, regulation of immune responses, and resistance to radiotherapy and chemotherapy through interactions with up- or downstream targeted molecules [154, 182]. In the treatment of common digestive tract tumors, radiotherapy and chemotherapy are pivotal. However, their effectiveness is limited. Targeting m6A regulators and their downstream targets shows promise as a molecular targeted therapy for advanced gastrointestinal tract cancers.[183–185]. Approximately 50% of patients with gastrointestinal stromal tumors (GIST) develop resistance to imatinib treatment within two years. Recent studies have revealed increased m6A modification in imatinib-resistant GIST cells and tissues. METTL3 upregulation leads to imatinib resistance by enhancing the expression of multidrug resistance protein 1 (MRP1). This leads to a reduction in the intracellular concentration of imatinib, promoting imatinib resistance in gastrointestinal stromal tumor cells [186]. METTL3 is also an effective target for improving conventional treatment efficacy in patients with pancreatic cancer. Knocking down METTL3 expression in pancreatic cancer cell lines enhances their sensitivity to anticancer radiotherapy and chemotherapeutics such as gemcitabine, cisplatin, and 5-fluorouracil [187]. Furthermore, inhibiting the METTL3-mediated activation of frizzled10 in liver cancer cells contributes to lenvatinib resistance by regulating the downstream β-catenin/c-Jun/Mitogen-activated protein kinase kinase (MEK)/ERK axis [98]. These findings suggest the potential for METTL3-targeted therapeutic strategies and provide new directions for personalized treatment of patients with pancreatic cancer.

Besides being a well-known m6A writer, ALKBH5 also collaborates with METTL14/IGF2BPs to suppress tumor cell glycolysis by negatively regulating the JMJD8/PKM2 signaling axis, thereby slowing down colorectal cancer progression. In preclinical tumor models, an ALKBH5 mRNA delivery system restored ALKBH5 levels at the tumor site, suppressing colorectal cancer growth and offering a novel clinical target [140]. Moreover, studies have found that RNA-binding protein IGF2BP1 binds to apoptotic protease-activating factor 1 (APAF1)-binding lncRNA (ABL) and recognizes METTL3-mediated m6A modification on ABL, maintaining the stability of ABL. This interaction between IGF2BP1 and ABL leads to gastric cancer cell resistance to apoptosis induced by chemotherapy such as 5‐fluorouracil and paclitaxel [188]. ALKBH5 has emerged as a promising candidate for drug development with its significant tumor-suppressive functions and the ability to sensitize pancreatic cancer cells to chemotherapy. It mediates m6A demethylation of DNA damage-inducible transcript 4 (DDIT4) and inhibits the methylation of WIF-1. This upregulates DDIT4 antisense RNA1, activating the Wnt and the mTOR signaling pathways, impairing the chemosensitivity of pancreatic cancer cells to gemcitabine [159, 189].

Currently, several targeted drug delivery systems and clustered regularly interspaced short palindromic repeats (CRISPR)/CRISPR-associated proteins systems have been successfully developed to regulate tumor m6A modification levels for anticancer purposes [190–193]. Engineered small extracellular vesicles were used to effectively deliver short interfering RNA targeting YTHDF1, leading to efficient depletion of YTHDF1 expression in gastric cancer tissues. This approach inhibited gastric cancer progression and metastasis by blocking the translation of frizzled7 and subsequently inactivating the Wnt/β-catenin pathway [194]. Furthermore, it also triggered a strong interferon-γ response, resulting in enhanced cytotoxic T lymphocyte response and tumor-associated macrophage-mediated phagocytosis [195]. These findings highlight the potential of targeting m6A regulators through RNA interference as a promising strategy for cancer treatment [196]. In addition, m6A modifications can also shape the immune landscape by influencing cytokine production, immune cell differentiation, and the inflammatory response, which are critical factors in cancer progression and response to therapy. Applying these insights to gastrointestinal cancers could reveal new mechanisms by which m6A modifications influence tumor immunity and help identify potential therapeutic targets for modulating the immune response to cancer [197].

However, while the current research on m6A modifications in gastrointestinal cancers has provided valuable insights, it is crucial to acknowledge the limitations and potential biases in recent research on m6A modifications in gastrointestinal tract cancers. One notable limitation is the relatively small sample sizes used in most clinical studies concerning the verification of clinical significance of targeting m6A modifications in gastrointestinal cancers. The majority of clinical studies of M6A in gastrointestinal tumors have an inadequate sample size, which may affect the reliability and generalizability of the findings. Additionally, the common use of different experimental models, such as gastrointestinal cancer cell lines versus animal models, may not accurately reflect in vivo biology and introduce variability and the difficulty of successful translation into a clinical setting for human patients. Moreover, there are differences among the methodologies used for the detection and analysis of the specific m6A modification in different studies. Overall, further studies with larger sample sizes, standardized methodologies, and comprehensive analyses are needed to fully understand the role of m6A modifications in these gastrointestinal tract cancers.

In summary, m6A modification has broad prospects in clinical diagnostics and treatment. Detecting m6A modification levels and implementing therapeutic strategies targeting m6A regulators and their downstream targets can enhance diagnostic accuracy and treatment outcomes in gastrointestinal tract cancers, ultimately improving clinical efficacy and patient outcomes.

## Conclusion

Increasing evidence consistently links alterations in m6A regulatory proteins and global m6A modification patterns with the occurrence and progression of gastrointestinal tract cancers. Multiple large-scale cohorts confirm the correlations between m6A level, patient prognosis, and diagnostic reliability, presenting m6A levels as promising indicators for both prognosis and diagnosis in these cancers. Dysregulation of m6A modification impacts the pathogenesis of tumor progression by regulating biological processes such as tumor cell proliferation, invasion, and metastasis. Given the critical roles of m6A methylation in several types of gastrointestinal tract cancers, m6A modification holds promise as a potential therapeutic target. However, significant research gaps remain, particularly in understanding how m6A regulators interact with other epigenetic factors and contribute to cancer heterogeneity. The development of precise biomarkers for m6A modification and effective therapeutic strategies targeting m6A-related pathways also necessitates further investigation. Addressing these unresolved challenges is crucial for advancing our knowledge and translating it into clinical applications that significantly improve patient outcomes. Future research should focus on elucidating the intricate molecular mechanisms of m6A modifications in gastrointestinal tract cancers and developing targeted therapies that leverage this knowledge.

## Data Availability

Not applicable.
